# The Determinants for Food Safety Push Notifications on Continuance Intention in an E-Appointment System for Public Health Medical Services: The Perspectives of UTAUT and Information System Quality

**DOI:** 10.3390/ijerph17218287

**Published:** 2020-11-09

**Authors:** Yu-Ping Lee, Hsin-Yeh Tsai, Athapol Ruangkanjanases

**Affiliations:** 1 Department of Business Administration, Shu-Te University, Hengshan Road, No. 59, Kaohsiung 82445, Taiwan; winona@stu.edu.tw (Y.-P.L.); hsinyeh@stu.edu.tw (H.-Y.T.); 2 Chulalongkorn Business School, Chulalongkorn University, 254 Phayathai Road, Bangkok 10330, Thailand

**Keywords:** push notifications of food safety, e-appointment, unified theory of the acceptance and use of technology (UTAUT), information system quality (ISQ), public medical services, partial least squares (PLS)

## Abstract

Compared to other appointment methods in public hospitals, registering through the Internet or utilizing e-appointments, or registering online as an outpatient, can provide more information to the user. This research investigated the integration of unified theory of the acceptance and use of technology and information system quality in determining factors that influence the adoption of e-appointments by patients, based on the requirements of food safety consultation in Taiwan. Empirical data from 369 valid samples were assessed using Partial Least Squares (PLS). The key findings of this study indicated that patients’ acceptance of e-appointments was influenced by users’ perceptions (i.e., performance expectancy and facilitating conditions), along with information quality and service quality. The practical and academic implications are provided for future practitioners and scholars, and to enhance patients’ usage of e-appointments in their healthcare activities.

## 1. Introduction

As information technology continues to advance, self-service technologies are replacing many of the manpower requirements for counter operations [1,2,3]. As a result, hospital-related medical services are also actively promoting online registration systems to patients. It is a common trend for businesses, even in the medical industry, to use the internet to provide services. The most common is the electronic appointment (e-appointment) provided by medical hospitals. E-appointments can be booked manually by telephone, automatically by telephone, or online [4,5]. Compared to other appointment methods in public hospitals, registering through the internet or for an e-appointment, or registering online as an outpatient, can provide more information to the user [4,6,7,8]. The most important information, including the type of disease and the doctor’s expertise, can be displayed on the hospital website. In addition, patients can follow the appointment status through real-time information on the website, including the number of appointments and the current situation of the clinic, so that people who go to the clinic can arrange their arrival time more flexibly. Unlike clinics where patients are admitted by family physicians before being hospitalized, hospitals admit and treat patients within the institution only. Patients have to make reservations with doctors before they can be diagnosed. Patients can either undertake on-site registration, or make an appointment in advance [9]. Waiting in line to be seen by a doctor at a medical site can be a lengthy process. In Taiwan, some prestigious doctors take only five on-site registrations per clinic time, and patients may wait for hours before the clinic begins.

On the other hand, in recent years, there have been frequent reports of major food safety incidents, which have been widely exaggerated by the mass media. Due to the food safety crisis, governments around the world have started to focus on the management of food safety from farm to fork. Patients with foodborne illnesses should be taken more seriously, and carefully managed. Besides the basic need for patients to eat a balanced diet, all hazardous substances need to be controlled; physical, chemical, and microbial contamination must be eliminated, and the patient’s dietary safety and knowledge must be enhanced in order for the patient to eat with peace of mind [10]. Food safety incidents can cause economic losses, social disruption, and also have an impact on the environment, so manufacturers involved in the food supply chain and relevant government departments are working to reduce the frequency and severity of their occurrence [11,12,13]. However, due to the public’s lack of proper food safety understanding and knowledge of chemistry, they are often unable to interpret the reports accurately, thus causing unnecessary panic at times. We hope that through e-appointments, the concepts of food safety and related knowledge can be effectively conveyed to the public, to promote knowledge so that users can read the reports correctly, judge the accuracy of the content, and at the same time implement this scientific knowledge in their lives.

Recently, Venkatesh et al. [14] developed a model based on the integration of eight information technology (IT) usage patterns: a unified theory of the acceptance and use of technology (UTAUT) was proposed. The study empirically examined the predictive effect of the model on the willingness to use technology, and tested the relevant user characteristics as moderators in this model. UTAUT is based on a systematic literature review from eight theoretical models and 32 constructs, to integrate four new determinants: performance expectancy, effort expectancy, social influence, and facilitating conditions, with four moderators (gender, age, experience, and voluntariness of use). Past studies have also found that UTAUTs have up to 70% explanatory power for usage behavior [14,15]. Other scholars, including Al-Gahtani et al. [16] and Schaper and Pervan [17], have tested the UTAUT on different samples and obtained the same results. Some studies have also extended the UTAUT model to examine willingness to use IT [18,19,20,21]. The extent to which the theory of user acceptance of new technological devices has been valued and flourished means that UTAUT research has been of great importance to the development of new technology. The implementation of information systems is of paramount importance. However, evidence related to e-appointments in medical institutions is still scarce. Thus, this research attempted to propose and evaluate the UTAUT and information system successful model for explaining e-appointment system adoption and its related issues in public medical services. The main purpose of this paper was twofold: first, to apply UTAUT of Venkatesh et al. [14] as the theoretical basis and propose a model for the characteristics of e-appointments to promote food safety information and knowledge, by testing a number of important pre-requisites that affect willingness to use e-appointments and food safety uncertainty. Second, this study extended UTAUT by considering information system quality, to explain and predict patients’ intentions around e-appointments with regards to food safety.

The rest of the article is organized as follows. Section 2 (Literature Review) illustrates previous research on the UTAUT and information system quality. Section 3 (System Description) describes the architecture and design of the proposed system. The developed system is outlined in Section 4. Suggestions and directions for further research are presented in the final section (Discussion and Conclusion).

## 2. Literature Review

### 2.1. Unified Theory of Acceptance and Use of Technology (UTAUT)

Many scholars have investigated the main factors influencing intention and behavior with respect to different technologies; however, these studies had been conducted from a single theoretical perspective. For example, the well-known technology acceptance model has been used to explain the influence of external variables on behavioral intentions [22]. Venkatesh et al. [14] recognized the inadequacy of using a unidimensional approach to understand individuals’ technology use intentions and behaviors. On the other hand, they also considered that each of these theoretical models has its own distinctive features and explanatory power, and thus proposed an integrated model of technology acceptance and use: UTAUT [14]. UTAUT integrated eight theoretical models and theories related to use intention and behavior, including the Theory of Reasoned Action [23], Technology Acceptance Model [22], Motivational Model [24], Theory of Planned Behavior [25], Combined TAM-TPB Model [26], Model of PC Utilization [27], Innovation Diffusion Theory [28,29], and Social Cognitive Theory [30,31], in an attempt to understand the acceptance of new technologies by thoroughly and comprehensively exploring the factors that influence individuals’ use intentions and behavior. The empirical results show that the model has a 70% better ability to explain the intentions and behaviors around use of information technology, which is significantly better than other theoretical models of technology acceptance.

In UTAUT, Behavior Intention (BI) is affected by performance expectancy, effort expectancy, social influence, and facilitating conditions. In addition, these factors are moderated by gender, age, experience, and voluntariness of use. UTAUT has been widely modified to fit into medical settings and different workplaces [32,33,34]. The four determinants of UTAUT are explained as follows:

- Performance expectancy: refers to the extent to which individuals believe that the use of IT can enhance their work performance.
- Effort expectancy: refers to the amount of effort that an individual must put into using the system. Information technologies will only be accepted and used if they have a good and user-friendly interactive interface, and an easy to operate system.
- Social influence: refers to the degree to which personal perception is important as to whether he or she should use the technology. In other words, an individual’s acceptance or use of information technology is somewhat influenced by their significant others.
- Facilitating conditions: refers to the level of support that an individual perceives from the organization and technology-related equipment, including the support of the computer hardware and software, or help in operating the technology or system.

Zhou et al. [35] proposed a modified UTAUT to illustrate that nurses’ adoption of hospital electronic information management systems, in terms of behavioral intention, was influenced by social influence and facilitating conditions. Maillet et al. [32] used the extended UTAUT to evaluate end-user acceptance and satisfaction as key factors for successful implementation of an Electronic Patient Record. Prior research has also not only confirmed the impacts of critical constructs from the original UTAUT model, but also identified perceived security as a new determinant that directly influences usage intentions in the home healthcare domain [36]. Despite the potential medical information services have in improving the quality of healthcare delivery services, few studies have been carried out on the adoption of e-appointments and push notifications for food safety. Therefore, this study attempts to adopt the basic constructs of UTAUT, and extend it based on the perspectives of information system quality.

### 2.2. Information System Quality

With the innovation and revolution of IT, from the physical environment to the internet economy, IT has been successfully applied to the consumer in many ways. The top level is the competitive advantage of an enterprise. Therefore, DeLone and McLean [37] argued that system quality and information quality will have a significant impact on the use and performance of information systems. Use and user satisfaction causes personal impact, and personal impact causes organizational impact. In order to bring the information system success model up to date, DeLone and McLean [38], in addition to the new service quality dimension added by Pitt et al. [39], added a new net benefit dimension, so that its application extends to a wide range of information system applications.

King and Epstein [40] examined 236 information systems and concluded the values of IS were cost efficiency, comparability, decision relevance, freedom from bias, information sufficiency, information understandability, reporting cycle, reporting delay, system reliability, and quantitativeness. Based on total quality management (TQM), Parasuraman et al. [41] proposed a conceptual model of service quality and refined it as service quality (SERVQUAL) in 1988, with five dimensions: tangibility, reliability, responsiveness, assurance, and empathy. They believed that the service quality customers feel depends on their perceived difference between their expectations and the services they receive. Customers tend to have higher satisfaction if the service quality they perceive exceeds their expectations. Miller and Doyle [42] measured the effectiveness of IS, and set up four indicators: completeness and accuracy of information, and relevance and timeliness of reports. Doll and Torkzadeh [43] developed a 12-item End-User Computing Satisfaction (EUCS) instrument, comprising five first-order latent variables: content, accuracy, format, ease of use, and timeliness. The construct “ease of use” was first found in their instrument.

In addition, the transmission of information to the patient requires a stable system to support this task. Traditionally, system quality measures have focused on the functional aspects of the system, such as system reliability, responsiveness, and flexibility. However, in the case of e-appointments, patients may expect not only the above factors, but also an easy-to-use or navigational interface, as well as a rapid response time [44]. Usability is the ability of a shopping site to provide an easy-to-use interface that allows customers to fulfill their specific purpose (e.g., product information). Reliability means that the shopping site’s system is stable (e.g., the system does not crash easily when you swipe your card online). In the case of a website, adaptability means that the site can dynamically adjust its strategy as customer needs change, and response time means that the site can respond quickly, with reasonable page load times. Chen and Yen [45] argued that a good website design is acceptable to consumers. Therefore, medical institutions should carefully consider patients’ feelings in order to provide them with a comfortable experience for medical services.

DeLone and McLean [44] developed an updated multidimensional success model of information systems, which integrates three determinants: system, information, and service quality.

## 3. Research Design

### 3.1. Development of Hypotheses and the Research Model

Performance expectancy was defined as the degree to which using an e-appointment service would provide benefits to users through push notifications of food safety information. Bawack and Kamdjoug [34] found performance expectancy had a significant impact on behavioral intention in clinician adoption of health information systems in developing countries. Related empirical research also showed that the adoption is more positive when users believe that the new information system/information technology (IS/IT) will improve their work/life performance [46,47,48]. From the above review, the following hypothesis is derived:

**Hypothesis 1** **(H1).** Performance expectancy has a positive impact on continuance intention.

Effort Expectancy was developed from the foundations of perceived ease-of-use and complexity (Venkatesh et al., 2003). This construct is based on a model of technology acceptance that uses effort expectancy to explain the user’s level of acceptance. The results of existing studies showed that the easier the system is to learn and the less effort that is required to use the IS/IT, the more positive the attitude of the user is towards adopting the IS/IT [49,50,51]. Based on the above evidence, Hypothesis 2 is consequently derived:

**Hypothesis 2** **(H2).** Effort expectancy has a positive impact on continuance intention.

Previous research has indicated social influence and facilitating conditions have positive influences on behavioral intention in clinician adoption of health information systems [34]. Sharifian et al. [52] argued that social influence and facilitating conditions were substantial in accounting for the behavioral intention to use hospital information systems. Prior research has also provided similar results regarding the relationship of social influence and facilitating conditions with behavioral intention [53]. From the above discussion, we derived the following two research hypotheses:

**Hypothesis 3** **(H3).** Social influence has a positive impact on continuance intention.

**Hypothesis 4** **(H4).** Facilitating Conditions have a positive impact on continuance intention.

The stability of a causal linkage associated with service quality influences customer evaluations and behavioral intention [54]. Empirical evidence has illustrated the positive linkage between information system quality and continuance intention [44,55,56,57,58]. Based upon the above literature, we proposed the following three hypotheses:

**Hypothesis 5** **(H5).** System Quality has a positive impact on continuance intention.

**Hypothesis 6** **(H6).** Information Quality has a positive impact on continuance intention.

**Hypothesis 7** **(H7).** Service Quality has a positive impact on continuance intention.

### 3.2. Measurement Items and Subjects

**Figure 1:**
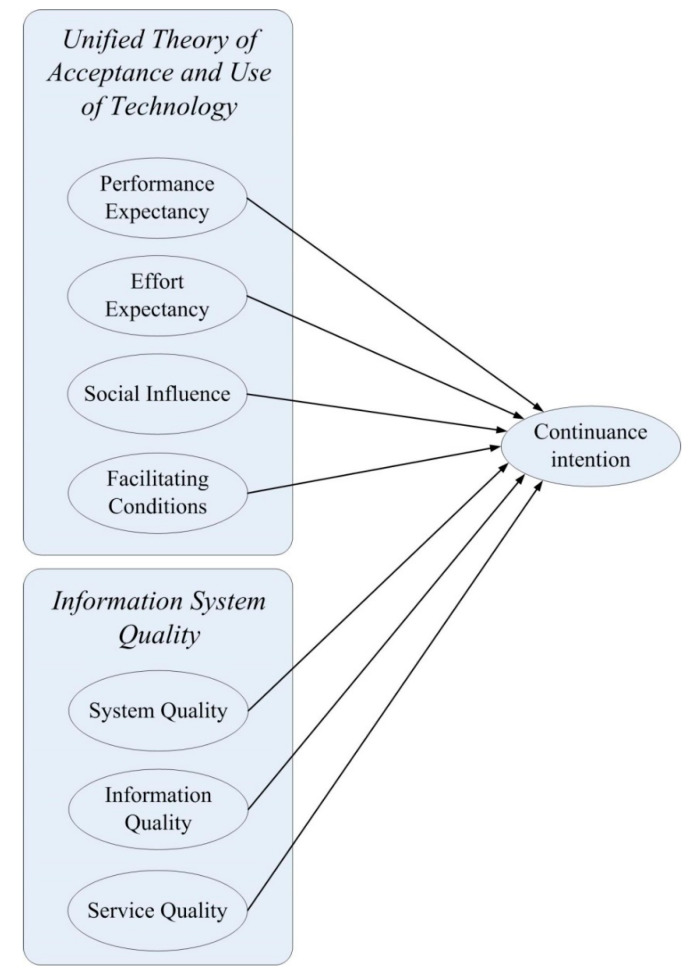
Research model.

Based on the UTAUT [14], three important latent variables of the Information Systems Success Model [44], including information quality, system quality, and service quality, were added to form the seven hypotheses of this study (Figure 1). All the questionnaire items were taken from the relevant literature and modified to fit the e-appointment context of this study. In order to ensure the reliability and validity of the measurement instrument, this questionnaire was pre-tested prior to its formal administration. Three experts (including two senior engineers of information systems in hospitals and one university professor) were asked to provide their opinions on the meaning and syntax of the questionnaire items, and to revise some of the wording in order to ensure validity of the questionnaire. All the questions in the research framework were on a seven-point Likert scale, from “strongly disagree” to “strongly agree”, for each scale item.

In this study, the questionnaires were collected from three public medical hospitals in Taiwan, and the samples were composed of patients and users who had issues related to dietary adjustment for various diseases, nutritional assessment, diet design and food selection and preparation, selection of special foods and nutritional supplements, nutritional counseling for various life stages, weight control, and body composition measurement and who used the e-appointment system to book nutritionists. In the field of health information services, e-appointments are probably the most relevant and popular health information system for health websites, providing a convenient alternative to in-person and telephone registration. The mobile app allows patients and users to log in to their personal information, and then select a medical option on the screen. After selecting a department, the e-appointment will generate a registered visit number and display it on the mobile app. In order to improve the quality and validity of the scale, a small sample was tested one month before the actual large-scale questionnaire was administered. Three outpatient physicians and 10 users with experience in using e-appointments were used as pre-test subjects in this study. Large-sample surveys were conducted by pre-testing and revising the prototype scale questionnaire in the first stage. This study was conducted by means of a questionnaire survey, which was distributed to users of the e-appointment system for a period of one month from 1 October 2018 to 31 October 2018. The questionnaires were collected and compiled after eliminating invalid questionnaires, giving a total of 369 valid samples. The basic information of the questionnaire was divided into seven categories: gender, age, education level, and the number of hospital visits per year. Statistical scales such as frequency distribution and percentages were used, and narrative statistical analysis was also conducted. The distribution of the sample structure was as follows: Male users accounted for 46.6% of the total sample, and female users accounted for 53.4%. In terms of education level, users with a university degree or above were the majority, accounting for about 45% of the sample. As for the occupation of the respondents, the majority were in the service sector, accounting for about 47% of the total (as shown in Table 1).

## 4. Data Analysis

In order to investigate the relationships between the variables and verify the validity of the research model, this study adopted Partial Least Squares (PLS), a structural equation model (SEM) analysis technique based on regression analysis, which is highly practical and better than the linear structural relationship model analysis technique, as it deals with both reflective and formative model structures [59,60]. The requirements for variables to conform to a normal distribution, randomness, and sample size are less stringent [61]. In addition, PLS can overcome multicollinearity problems, effectively handle interference data and missing values, and has good prediction and interpretation capabilities. The sample size of this study was small, but PLS is not limited by sample size and variable allocation patterns. The number of bootstrap resamplings was 1000 times [62] to ensure the stability of the estimation of each variable. In this study, SmartPLS (Boenningstedt, Germany) developed by Ringle et al. [63] was used as the analysis tool. The analysis and estimation of PLS were divided into two stages. The first stage was to analyze the confidence and validity of the measured model, while the second stage was to estimate and validate the coefficients of the path and diameter of the structural model, and the explanatory power of the model. The software tool we used was SmartPLS 3.3.2, which is based the least square method for the analysis. Therefore, this study divided the research model into two stages of examination; the first stage was to evaluate the measurement model, and the second stage was to perform the structural model analysis [64].

### 4.1. Measurement Model

According to Hair et al. [65], the following four criteria were followed in this study: (1) the assessment of the factor loadings of the measurement variables on the latent variable; the recommended value for a good individual item factor loading should be greater than 0.5; (2) the composite reliability of the potential variables is a test of the composition of the insistency of all the measurement variables within a latent variable; the recommended value for a good individual item level should be greater than 0.7; (3) the extraction variance of the latent variable is the average variance explained (AVE) by the measurement items of the latent variable; the recommended value for each latent variable should be greater than 0.5; and (4) the loadings of the measurement items on the latent variable should be significant. The analysis results showed that all the measurement items were adequate and significant. The composite reliability of each latent variable in this questionnaire was above 0.7, and the AVE of all latent variables was at least greater than 0.5, which also met the evaluation standard from Fornell and Larcker (1981) and Hair et al. (2010). According to the analysis results in Table 2, the reliability and convergent validity of this questionnaire meet the above four requirements. In addition, we checked the discriminant validity based on the guidelines from Fornell and Larcker [66]. As shown in Table 3, the square root of the AVE (diagonal elements) should be higher than corresponding off-diagonal elements (i.e., correlations).

We further compared the factor loadings and cross-loadings to estimate the discriminant validity of the measurement items employed in testing the measurement model [62]. Table 4 presents the factor loadings and cross-loadings of all reflective measures in the proposed model, and shows that the factor loadings for each construct are greater than cross loadings. Therefore, our measurement model satisfied the discriminant validity standard suggested by Chin [62].

### 4.2. Structural Model

In this study, PLS was used as a statistical procedure and SmartPLS as an analytical tool to analyze the strength and direction of the relationships between the latent variables in the structural model. The path coefficients represented the strength and direction of the relationships between the studied components. The hypotheses were verified to be valid if checked for significance, and consistent with the expected direction of the hypothesis. The results of the analysis of the structural model in this study are shown in Table 5.

## 5. Conclusions

### 5.1. Research Findings and Implications

As the accessibility of the internet increases, e-appointment systems have become an essential service in the hospitals and medical services, including to manage push notifications for food safety. E-appointment systems are a type of hospital outpatient pre-register appointment system operating through the internet. Since the system utilizes self-service technology, there is no online personnel available if the user encounters difficulties in operations. User acceptance and the quality of the e-appointment system are vital for the continuance intention of users. In order to improve the continued use of the user, our recommendations are as follows:

(1) Performance expectancy: The e-appointment system must have several advantages over traditional methods, such as time-saving quality or effectiveness, so that user willingness to reuse the system will be increased. For example, the e-appointment could send patients a message one day before the appointment as a reminder, or the system could provide a reliable estimated waiting time so patients can plan their trip to the hospital. An e-appointment system could also be used for push notifications of food safety, and give reminders related to nutrition. Refining the functions of the e-appointment system will improve performance expectancy.

(2) Service quality: System administrators should provide services, not just e-appointment functions. Relevant services, such as bulletin Q&As, health information, or even online patient services, will encourage patients and their families to use the e-appointment system repeatedly.

(3) Facilitating conditions: the e-appointment system could be accessed using either computers or handheld devices. Therefore, the hospital must ensure the e-appointment system is compatible with any device that connects to it. User interfaces for computers and smart phones are quite different. System administrators must take this into consideration.

(4) Information quality: the e-appointment system should provide not only registration functions, but also information on different medical services. Web pages of the hospital should meet user needs, and the contents should be updated regularly. These practices will contribute to the promotion of information quality.

### 5.2. Conclusions

Hospitals can gain a competitive advantage by improving the quality of their services and their relationship with their patients. In an increasingly competitive healthcare market, the quality of a hospital’s services and the quality of its relationships with patients are key success factors. Service quality is essential for hospitals to maintain good relationships with patients. For first-time patients booking a nutritionist to evaluate food safety information, the e-appointment system provides them with their first impression of hospital service quality. If the hospital information system can deliver adequate services to patients, higher user satisfactory can be produced. In addition, if more people use the e-appointment system, physicians and nutritionists can reduce the number of employees working on-site or taking telephone registrations. Therefore, an e-appointment system could not only improve the efficiency of hospital registration procedures, but also reduce labor costs of the hospital. This study incorporates UTAUT and ISQ to explore the continuous user behaviors relating to an e-appointment system. The results of this study may be helpful for hospital administrators to refine their e-appointment system.

According to the hypothesis testing, the results of this study are as follows. First, among the factors that impact user intentions around a hospital e-appointment system, performance expectancy has the greatest effect, followed by service quality, facilitating conditions, and information quality. It can be concluded that performance expectation, or usefulness, is a critical factor to increase user intention relating to hospital e-appointment systems. Patients believe using an e-appointment system is more efficient and can reduce waiting times, while also obtaining up-to-date food safety information while registering and recovering. In other words, an e-appointment system can help patients and their families to speed up the registration process. This will lead e-appointment users to use the system continuously. These findings are in agreement with those of Luarn and Lin [67].

Secondly, because users can always access the e-appointment system through the Internet to update their registration information or to check available times for physicians and nutritionists, the hospital information system plays a critical role in terms of the quality of service. Except in an emergency, patients cannot be treated without being registered. An e-appointment system in medical services is a bridge between patients, physicians, nutritionists, and the hospital. The service quality of the e-appointment system will affect the user intention for continuous usage. If the operation interface of the system is well designed and can provide 24/7 services, patients will have no reason to reject the system and will have a higher intention to use it.

A third factor affecting user intention to use the e-appointment system is facilitating conditions, which refers to the resources users have, such as the internet or devices, to access it. In addition, the compatibility of user devices and the e-appointment system, or the users’ computing proficiency, will also affect user intention toward the e-appointment system.

The last factor that impacts user intention for using an e-appointment system is information quality. If the e-appointment system provided correct and precise information for users to complete registration quickly, or provided users with diagnostic progress, this makes the system more valuable and increases user intention to reuse the system.

These four factors are vital to promote the continuous usage intention of an e-appointment system. However, in this study, website expectation, social impact, and system quality did not have as significant of an impact on the user intention toward reuse of the e-appointment system. The reason for this may be that the e-appointment system has been optimized so it is easy to operate and understand, and is able to complete the registration process quickly. Therefore, effort expectations do not affect the user intention for reuse of the system. That is, the ease of use of the system is no longer a significant influence on the continuous usage intention of the e-appointment system.

In addition, e-appointment system users are most likely Internet surfers. They use the system even when not encouraged to do so by the others; therefore, the social influence is minimal. The system quality has little influence on the behavior intention, meaning the e-appointment system is stable, reliable, and well designed. Therefore, SYQ does not become a significant factor in enhancing the user intention to use the system again.

### 5.3. Research Limitations and Suggestions for Future Research

The samples in this study were users with e-appointment experience. Future research could compare users with or without e-appointment experience, to understand the needs of different patients and their families. For example, users with less internet experience might not be familiar with the e-appointment system, and need more online assistance. However, experienced internet users have short learning curves and tend to search information in the hospital website by themselves. Configuration of the e-appointment system for different levels of internet users will impact user willingness to reuse the system. Venkatesh et al. [14] found that effort expectancy, social influence, and facilitating conditions were regulated by the degree of user computing experience. Future studies may distinguish users with different networking proficiencies and provide them with different levels of internet services, for example, by providing smart phone users with a hospital e-appointment app.

## Figures and Tables

**Table 1:** Sample structure.

Characteristics	Frequency	Percent (%)
*Gender*		
Male	172	46.6%
Female	197	53.4%
*Age*		
Under 25	28	7.6%
26–35	50	13.6%
36–45	124	33.5%
46–55	69	18.7%
Over 55	98	26.6%
*Education level*		
High school certificate or below	97	26.3%
Technical school	68	18.4%
Undergraduate degree	166	45.0%
Master or higher degree	38	10.3%
*Occupation*		
Student	38	10.3%
Service	173	46.9%
Manufacturing	77	20.8%
Others	81	22.0%
*Frequency of medical visits per year*		
Under 1 time	82	22.2%
1–3 times	168	45.5%
Over 3 times	119	32.3%

**Table 2:** Assessment of the measurement model.

Construct	Indicator	Mean	Std. Dev.	Factor Loading	*t*-Value	CR	AVE
PE	PE1	6.382	0.791	0.842	32.968	0.914	0.780
	PE2	6.564	0.656	0.912	50.277		
	PE3	6.523	0.654	0.895	52.678		
EE	EE1	5.957	0.825	0.850	31.776	0.908	0.767
	EE2	5.992	0.921	0.861	34.141		
	EE3	5.981	0.866	0.914	88.699		
SI	SI1	5.114	1.402	0.927	41.711	0.910	0.835
	SI2	5.425	1.301	0.900	28.772		
FC	FC1	6.222	0.789	0.874	40.193	0.900	0.751
	FC2	6.247	0.831	0.902	62.785		
	FC3	6.146	1.072	0.822	21.744		
SYQ	SYQ1	6.095	0.736	0.896	56.824	0.905	0.761
	SYQ2	5.951	0.873	0.866	35.894		
	SYQ3	5.995	0.823	0.855	39.443		
IQ	IQ1	6.230	0.683	0.778	26.785	0.925	0.712
	IQ2	6.241	0.922	0.787	23.265		
	IQ3	6.103	0.859	0.858	42.817		
	IQ4	6.114	0.825	0.886	54.878		
	IQ5	6.130	0.839	0.903	72.620		
SERQ	SERQ1	5.738	0.682	0.749	21.136	0.924	0.753
	SERQ2	5.994	0.928	0.872	55.672		
	SERQ3	5.771	0.852	0.917	65.087		
	SERQ4	5.724	0.825	0.922	103.816		
CI	CI1	0.671	1.573	0.870	49.618	0.925	0.805
	CI2	0.735	4.141	0.912	54.645		
	CI3	0.827	2.721	0.908	82.731		

Note 1: CR= Composite Reliability; AVE = Average Variance Extracted. Note 2: PE = Performance Expectancy; EE = Effort Expectancy; SI = Social Influence; FC = Facilitating Condition; SYQ = System Quality; IQ = Information Quality; SERQ = Service Quality; Continuance Intention.

**Table 3:** Correlation matrix and discriminant validity.

	PE	EE	SI	FC	SYQ	IQ	SERQ	CI
PE	***0.883***							
EE	0.427	***0.876***						
SI	0.204	0.374	***0.914***					
FC	0.411	0.654	0.315	***0.867***				
SYQ	0.435	0.537	0.343	0.413	***0.872***			
IQ	0.528	0.631	0.306	0.518	0.687	***0.844***		
SERQ	0.401	0.519	0.349	0.458	0.763	0.706	***0.868***	
CI	0.553	0.531	0.261	0.538	0.584	0.642	0.651	***0.897***

Note 1: Diagonal elements in bold and italics are square roots of the average variance extracted. Note 2: PE = Performance Expectancy; EE = Effort Expectancy; SI = Social Influence; FC = Facilitating Condition; SYQ = System Quality; IQ = Information Quality; SERQ = Service Quality; Continuance Intention.

**Table 4:** Standardized factor loadings and cross-loadings for measures.

	PE	EE	SI	FC	SYQ	IQ	SERQ	CI
PE1	***0.842***	0.388	0.201	0.357	0.363	0.433	0.340	0.474
PE2	***0.912***	0.356	0.141	0.361	0.376	0.478	0.336	0.496
PE3	***0.895***	0.388	0.200	0.37	0.415	0.487	0.385	0.494
EE1	0.363	***0.850***	0.309	0.519	0.428	0.480	0.426	0.37
EE2	0.378	***0.861***	0.308	0.514	0.478	0.559	0.433	0.449
EE3	0.382	***0.914***	0.359	0.664	0.497	0.604	0.497	0.543
SI1	0.155	0.328	***0.927***	0.302	0.300	0.272	0.320	0.255
SI2	0.222	0.357	***0.900***	0.273	0.330	0.290	0.318	0.219
FC1	0.387	0.586	0.290	***0.874***	0.395	0.444	0.410	0.465
FC2	0.404	0.577	0.293	***0.902***	0.351	0.467	0.400	0.501
FC3	0.268	0.536	0.232	***0.822***	0.328	0.435	0.382	0.428
SYQ1	0.400	0.523	0.305	0.396	***0.896***	0.655	0.630	0.508
SYQ2	0.339	0.421	0.311	0.368	***0.866***	0.549	0.618	0.473
SYQ3	0.397	0.458	0.283	0.321	***0.855***	0.591	0.740	0.541
IQ1	0.472	0.597	0.205	0.499	0.517	***0.778***	0.483	0.521
IQ2	0.409	0.410	0.265	0.387	0.495	***0.787***	0.540	0.456
IQ3	0.442	0.580	0.284	0.427	0.603	***0.858***	0.615	0.563
IQ4	0.461	0.505	0.248	0.431	0.636	***0.886***	0.639	0.575
IQ5	0.445	0.559	0.291	0.442	0.632	***0.903***	0.687	0.583
SERQ1	0.265	0.345	0.257	0.264	0.684	0.477	***0.749***	0.439
SERQ2	0.393	0.509	0.308	0.397	0.722	0.665	***0.872***	0.548
SERQ3	0.361	0.437	0.328	0.443	0.610	0.626	***0.917***	0.625
SERQ4	0.362	0.500	0.314	0.457	0.664	0.665	***0.922***	0.623
CI1	0.473	0.496	0.221	0.481	0.513	0.545	0.547	***0.870***
CI2	0.495	0.428	0.235	0.444	0.510	0.530	0.549	***0.912***
CI3	0.517	0.502	0.245	0.518	0.544	0.645	0.646	***0.908***

Note 1: The values of bold and italics are standardized loading loadings; the others are cross-loadings. Note: PE = Performance Expectancy; EE = Effort Expectancy; SI = Social Influence; FC = Facilitating Condition; SYQ = System Quality; IQ = Information Quality; SERQ = Service Quality; Continuance Intention.

**Table 5:** Hypothesis testing results.

Hypothesis	Path Direction	Path Coefficient	*t*-value	Result
H1	PE → CI	0.242 ***	4.317	Supported
H2	EE → CI	0.035	0.660	Not supported
H3	SI → CI	−0.033	0.934	Not supported
H4	FC → CI	0.182 *	2.099	Supported
H5	SYQ → CI	0.148 *	2.341	Not supported
H6	IQ → CI	0.048	0.777	Supported
H7	SERQ → CI	0.323 ***	5.131	Supported

Note 1: PE = Performance Expectancy; EE = Effort Expectancy; SI = Social Influence; FC = Facilitating Condition; SYQ = System Quality; IQ = Information Quality; SERQ = Service Quality; Continuance Intention. Note 2: * *p*-value < 0.05; ** *p*-value < 0.01; *** *p*-value < 0.005.

## References

[1] Chen S.C., Chen H.H., Chen M.F. (2009). Determinants of satisfaction and continuance intention towards self-service technologies. Ind. Manag. Data Syst..

[2] Lin J.S.C., Hsieh P.L. (2011). Assessing the self-service technology encounters: Development and validation of SSTQUAL scale. J. Retail..

[3] Horvath M.M., Rusincovitch S.A., Brinson S., Shang H.C., Evans S., Ferranti J.M. (2014). Modular design, application architecture, and usage of a self-service model for enterprise data delivery: The Duke Enterprise Data Unified Content Explorer (DEDUCE). J. Biomed. Inform..

[4] Chen S.C., Jong D., Lai M.T. (2014). Assessing the relationship between technology readiness and continuance intention in an E-appointment system: Relationship quality as a mediator. J. Med. Syst..

[5] Kitsios F., Stefanakakis S., Kamariotou M., Dermentzoglou L. (2019). E-service Evaluation: User satisfaction measurement and implications in health sector. Comput. Stand. Interfaces.

[6] Chen S.C., Liu S.C., Li S.H., Yen D.C. (2013). Understanding the mediating effects of relationship quality on technology acceptance: An empirical study of e-appointment system. J. Med. Syst..

[7] Zhang M., Zhang C., Sun Q., Cai Q., Yang H., Zhang Y. (2014). Questionnaire survey about use of an online appointment booking system in one large tertiary public hospital outpatient service center in China. BMC Med. Inform. Decis. Mak..

[8] Chang M.Y., Pang C., Tarn J.M., Liu T.S., Yen D.C. (2015). Exploring user acceptance of an e-hospital service: An empirical study in Taiwan. Comput. Stand. Interfaces.

[9] Vickers K.S., Ridgeway J.L., Hathaway J.C., Egginton J.S., Kaderlik A.B., Katzelnick D.J. (2013). Integration of mental health resources in a primary care setting leads to increased provider satisfaction and patient access. Gen. Hosp. Psychiatry.

[10] Lee L.A., Burks A.W. (2006). Food allergies: Prevalence, molecular characterization, and treatment/prevention strategies. Annu. Rev. Nutr..

[11] Wang J., Diao H., Tou L. (2019). Research on the influence mechanism of rational consumers’ food Safety supervision satisfaction. Int. J. Environ. Res. Public Health.

[12] Cho T.J., Kim S., Kim H.W., Park S.M., Rhee M.S. (2019). Changes in consumers’ food purchase and transport behaviors over a decade (2010 to 2019) following health and convenience food trends. Int. J. Environ. Res. Public Health.

[13] Lin P., Tsai H., Ho T. (2020). Food Safety Gaps between Consumers’ Expectations and Perceptions: Development and Verification of a Gap-Assessment Tool. Int. J. Environ. Res. Public Health.

[14] Venkatesh V., Morris M.G., Davis G.B., Davis F.D. (2003). User acceptance of information technology: Toward a unified view. MIS Q..

[15] Carlsson C., Walden P., Bouwman H. (2006). Adoption of 3G+ services in Finland. Int. J. Mob. Commun..

[16] Al-Gahtani S.S., Hubona G.S., Wang J. (2007). Information technology (IT) in Saudi Arabia: Culture and the acceptance and use of IT. Inf. Manag..

[17] Schaper L.K., Pervan G.P. (2007). ICT and OTs: A model of information and communication technology acceptance and utilisation by occupational therapists. Int. J. Med. Inform..

[18] Hoque R., Sorwar G. (2017). Understanding factors influencing the adoption of mHealth by the elderly: An extension of the UTAUT model. Int. J. Med Inform..

[19] Kalavani A., Kazerani M., Shekofteh M. (2018). Acceptance of evidence based medicine (EBM) databases by Iranian medical residents using unified theory of acceptance and use of technology (UTAUT). Health Policy Technol..

[20] Lin X., Wu R., Lim Y.T., Han J., Chen S.C. (2019). Understanding the Sustainable Usage Intention of Mobile Payment Technology in Korea: Cross-Countries Comparison of Chinese and Korean Users. Sustainability.

[21] Wang H., Tao D., Yu N., Qu X. (2020). Understanding consumer acceptance of healthcare wearable devices: An integrated model of UTAUT and TTF. Int. J. Med. Inform..

[22] Davis F.D., Bagozzi R.P., Warshaw P.R. (1989). User acceptance of computer technology: A comparison of two theoretical models. Manag. Sci..

[23] Fishbein M., Ajzen I. (1975). Belief, Attitude, Intention, and Behavior: An Introduction to Theory and Research, Reading.

[24] Davis F.D., Bagozzi R.P., Warshaw P.R. (1992). Extrinsic and intrinsic motivation to use computers in the workplace. J. Appl. Soc. Psychol..

[25] Ajzen I. (1991). The theory of planned behavior. Organ. Behav. Hum. Decis. Process..

[26] Taylor S., Todd P.A. (1995). Understanding information technology usage: A test of competing models. Inf. Syst. Res..

[27] Thompson R.L., Higgins C.A., Howell J.M. (1991). Personal computing: Toward a conceptual model of utilization. MIS Q..

[28] Rogers E.M. (1962). Diffusion of Innovations.

[29] Moore G.C., Benbasat I. (1991). Development of an instrument to measure the perceptions of adopting an information technology innovation. Inf. Syst. Res..

[30] Bandura A. (1986). Social Foundations of Thought and Action: A Social Cognitive Theory, 1st, Englewood Cliffs.

[31] Compeau D.R., Higgins C.A. (1995). Application of social cognitive theory to training for computer skills. Inf. Syst. Res..

[32] Maillet É., Mathieu L., Sicotte C. (2015). Modeling factors explaining the acceptance, actual use and satisfaction of nurses using an Electronic Patient Record in acute care settings: An extension of the UTAUT. Int. J. Med. Inform..

[33] Hsieh H.L., Kuo Y.M., Wang S.R., Chuang B.K., Tsai C.H. (2017). A study of personal health record user’s behavioral model based on the PMT and UTAUT integrative perspective. Int. J. Environ. Res. Public Health.

[34] Bawack R.E., Kamdjoug J.R.K. (2018). Adequacy of UTAUT in clinician adoption of health information systems in developing countries: The case of Cameroon. Int. J. Med. Inform..

[35] Zhou L.L., Owusu-Marfo J., Antwi H.A., Antwi M.O., Kachie A.D.T., Ampon-Wireko S. (2019). Assessment of the social influence and facilitating conditions that support nurses’ adoption of hospital electronic information management systems (HEIMS) in Ghana using the unified theory of acceptance and use of technology (UTAUT) model. BMC Med Inform. Decis. Mak..

[36] Alaiad A., Zhou L., Koru G. (2014). An exploratory study of home healthcare robots adoption applying the UTAUT model. Int. J. Healthc. Inf. Syst. Inform..

[37] DeLone W.H., McLean E.R. (1992). Information systems success: The quest for the dependent variable. Inf. Syst. Res..

[38] Delone W.H., Mclean E.R. (2004). Measuring e-commerce success: Applying the DeLone & McLean information systems success model. Int. J. Electron. Commer..

[39] Pitt L.F., Watson R.T., Kavan C.B. (1995). Service quality: A measure of information systems effectiveness. MIS Q..

[40] King W.R., Epstein B.J. (1983). Assessing information system value: An experimental study. Decis. Sci..

[41] Parasuraman A., Zeithaml V.A., Berry L.L. (1985). A Conceptual Model of Service Quality and Its Implications for Future Research. J. Mark..

[42] Miller J., Doyle B.A. (1987). Measuring the effectiveness of computer-based information systems in the financial services sector. MIS Q..

[43] Doll W.J., Torkzadeh G. (1988). The measurement of end-user computing satisfaction. MIS Q..

[44] Delone W.H., McLean E.R. (2003). The DeLone and McLean model of information systems success: A ten-year update. J. Manag. Inf. Syst..

[45] Chen K., Yen D.C. (2004). Improving the quality of online presence through interactivity. Inf. Manag..

[46] Chiu C.M., Wang E.T. (2008). Understanding Web-based learning continuance intention: The role of subjective task value. Inf. Manag..

[47] Chou S.W., Min H.T., Chang Y.C., Lin C.T. (2010). Understanding continuance intention of knowledge creation using extended expectation–confirmation theory: An empirical study of Taiwan and China online communities. Behav. Inf. Technol..

[48] Tam C., Santos D., Oliveira T. (2020). Exploring the influential factors of continuance intention to use mobile Apps: Extending the expectation confirmation model. Inf. Syst. Front..

[49] Escobar-Rodríguez T., Carvajal-Trujillo E. (2014). Online purchasing tickets for low cost carriers: An application of the unified theory of acceptance and use of technology (UTAUT) model. Tour. Manag..

[50] Tavares J., Oliveira T. (2016). Electronic health record patient portal adoption by health care consumers: An acceptance model and survey. J. Med. Internet Res..

[51] Baydas O., Goktas Y. (2017). A model for preservice teachers’ intentions to use ICT in future lessons. Interact. Learn. Environ..

[52] Sharifian R., Askarian F., Nematolahi M., Farhadi P. (2014). Factors influencing nurses’ acceptance of hospital information systems in Iran: Application of the Unified Theory of Acceptance and Use of Technology. Health Inf. Manag. J..

[53] Nikou S.A., Economides A.A. (2017). Mobile-based assessment: Investigating the factors that influence behavioral intention to use. Comput. Educ..

[54] Chiu C.K. (2009). Understanding relationship quality and online purchase intention in e-tourism: A qualitative application. Qual. Quant..

[55] Lee H., Kim J., Kim J. (2007). Determinants of success for application service provider: An empirical test in small businesses. Int. J. Hum. Comput. Stud..

[56] Ramayah T., Ahmad N.H., Lo M.C. (2010). The role of quality factors in intention to continue using an e-learning system in Malaysia. Procedia Soc. Behav. Sci..

[57] Motaghian H., Hassanzadeh A., Moghadam D.K. (2013). Factors affecting university instructors’ adoption of web-based learning systems: Case study of Iran. Comput. Educ..

[58] Stefanovic D., Marjanovic U., Delić M., Culibrk D., Lalic B. (2016). Assessing the success of e-government systems: An employee perspective. Inf. Manag..

[59] Urbach N., Ahlemann F. (2010). Structural equation modeling in information systems research using partial least squares. J. Inf. Technol. Theory Appl..

[60] Hair J.F., Hult G.T.M., Ringle C., Sarstedt M. (2016). A Primer on Partial Least Squares Structural Equation Modeling (PLS-SEM).

[61] Chin W.W., Newsted P.R. (1995). The importance of specification in causal modeling: The case of end-user computing satisfaction. Inf. Syst. Res..

[62] Chin W.W. (1998). The partial least squares approach to structural equation modeling. Mod. Methods Bus. Res..

[63] Ringle C.M., Wende S., Becker J.M. SmartPLS 3. Boenningstedt: SmartPLS GmbH. http://www.smartpls.com.

[64] Anderson J.C., Gerbing D.W. (1988). Structural equation modeling in practice: A review and recommended two-step approach. Psychol. Bull..

[65] Hair Jr J.F., Black W.C., Babin B.J., Anderson R.E. (2010). Multivariate Data Analysis: A Global Perspective.

[66] Fornell C., Larcker D.F. (1981). Evaluating structural equation models with unobservable variables and measurement error. J. Mark. Res..

[67] Luarn P., Lin H.H. (2005). Toward an understanding of the behavioral intention to use mobile banking. Comput. Hum. Behav..

